# Is antibiotic prophylaxis mandatory after the insertion of levonorgestrel-releasing intrauterine systemin order to decrease the risk of pelvic inflammatory disease?

**Published:** 2013-12-25

**Authors:** O Munteanu, L Radulescu, O Bodean, C Cirstoiu, D Secara, M Cirstoiu

**Affiliations:** * Department of Obstetrics and Gynecology, Bucharest University Emergency Hospital; ** Department of Biochemistry, “Carol Davila” University of Medicine and Pharmacy, Bucharest; *** Department of Orthopedics and Traumatology, Bucharest University Emergency Hospital

**Keywords:** levonorgestrel-releasing, intrauterine system, antibiotic prophylaxis, pelvic inflammatory disease

## Abstract

Abstract

Objective: This study was undertaken in order to determine if antibiotic prophylaxis is mandatory, after the insertion of levonorgestrel-releasing intrauterine system in order to decrease the risk of pelvic inflammatory disease.

Materials and methods: We prospectively evaluated 44 patients, admitted in the Bucharest Emergency Hospital between the 1ⁱ of February 2012 and the 1ⁱ of October 2012, in whom the levonorgestrel-releasing intrauterine system was inserted. The patients enrolled were divided into two groups. In group A, a number of 22 patients, received, after the insertion of levonorgestrel-releasing intrauterine system, 875mg Amoxicillin Trihydrate + 125 mg Potassium Clavulanate, a dose every 12 hours for 5 days. Group B was represented by the other 22 patients who did not receive antibiotic prophylaxis. All patients were reevaluated at 4 and 12 weeks after the insertion of levonorgestrel-releasing intrauterine system.

Results: During the first 4 weeks after the insertion of levonorgestrel-releasing intrauterine system only two patients, one from group A and one from group B were diagnosed with pelvic inflammatory disease. At a second follow up visit – 12 weeks after the insertion of levonorgestrel-releasing intrauterine system, no other patient was diagnosed with pelvic inflammatory disease.

Conclusion: Antibiotic prophylaxis is not mandatory, after the insertion of levonorgestrel-releasing intrauterine system in order to decrease the risk of pelvic inflammatory disease.

## Introduction

An intrauterine device (IUD) is a small polyethylene device, which contains copper or progesterone. IUDs are a form of long-acting reversible method of contraception, which is the most effective type of reversible birth control [**1**]. Also, IUDs are among the most cost-effective options for reversible contraception [**2**]. Therefore, IUDs are one of the most used and preferred methods of contraception due to their efficacy, reversibility and long term use [**5**-**3**]. 

The levonorgestrel-releasing intrauterine system is the most effective IUD available, with a 0.2% pregnancy rate at 1 year of use [**6**,**7**]. The levonorgestrel-releasing intrauterine system consists of a T-shaped polyethylene frame with a hormone reserve that contains 52 mg of levonorgestrel. The levonorgestrel is initially released at a rate of 20 ug/day and decreases to 11 ug/day after 5 years [**6**,**8**]. 

It seems that the mechanism of action of the levonorgestrel-releasing intrauterine system is multifactorial. The levonorgestrel induces an atrophy of the endometrial glands but it also changes the characteristics of the cervical mucus in order to prevent pregnancy [**6**,**9**]. Plus, the IUD modifies the physiological movement of spermatozoa and zygote, preventing nidation [**10**]. 

The most common side effects of levonorgestrel-releasing intrauterine system are amenorrhea, spotting and pain [**6**,**10**]. The last is a frequent symptom of pelvic inflammatory disease, one of the most redundant complications of patients with levonorgestrel-releasing intrauterine system [**11**,**12**]. 

## Objective

This study was undertaken in order to determine if antibiotic prophylaxis is mandatory, after the insertion of levonorgestrel-releasing intrauterine system in order to decrease the risk of pelvic inflammatory disease.

## Materials and methods

We prospectively evaluated 44 patients, admitted in Bucharest Emergency Hospital between the 1ⁱ of February 2012 and the 1ⁱ of October 2012, at whom levonorgestrel-releasing intrauterine system was inserted. All patients were informed of the advantages, disadvantages and possible side effects of this type of contraception. An informed consent was then taken from all the subjects of the study. 

At a visit prior to IUD insertion a preliminary evaluation was performed – all patients underwent a physical and echo-graphical examination, a PAP smear, screening for common sexually transmitted infections and a complete blood count was sampled. In all subjects a urine pregnancy test was performed – it was negative in all cases. Following clinical, paraclinical or imagistic criteria all patients suspected to have pelvic inflammatory disease were excluded. 

The insertion of levonorgestrel-releasing intrauterine system was performed by a experienced physician during the first 7 days of the menstrual cycle.

The patients enrolled were divided into two groups. In group A, a number of 22 patients received 875mgAmoxicillin Trihydrate+ 125 mg Potassium Clavulanate, a dose every 12 hours for 5 days after the insertion of levonorgestrel-releasing intrauterine system. Group B was represented by the other 22 patients who did not receive antibiotic prophylaxis. All the patients were reevaluated at 4 and 12 weeks after the insertion of levonorgestrel-releasing intrauterine system. The statistical analysis was conducted by using SPSS version 19. 

## Results

The global mean age of the evaluated patients was 28.31 years, with a mean age of 29.22 years in group A and of 27.4 years for group B.

 Of the 44 subjects, 37 (19 from group A and 18 from group B) were first-time users of levonorgestrel-releasing intrauterine system.

**Fig. 1:**
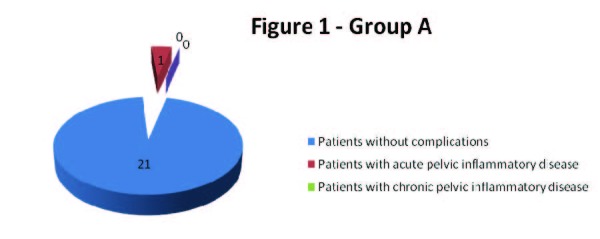
Group A

**Fig. 2:**
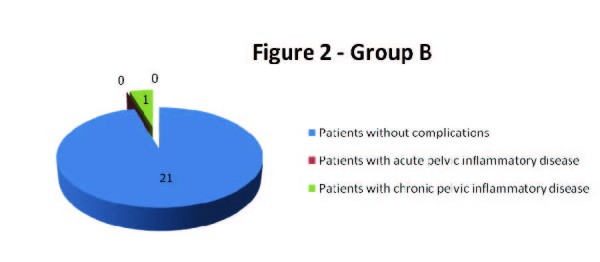
Group B

During the first 4 weeks after the insertion of levonorgestrel-releasing intrauterine system only two patients, one from group A and one from group B were diagnosed with pelvic inflammatory disease. The patient from group A, a first-time user of levonorgestrel-releasing intrauterine system accused severe lower abdominal pain after 14 days past insertion and following clinical and paraclinical criteria she was diagnosed with acute pelvic inflammatory disease; the IUD was extracted and she received specific antibiotic and anti-inflammatory treatment. However, the patient from group B experienced mild pain and leucorrhoea - at the follow up visit after 4 weeks past insertion she was diagnosed with chronic pelvic inflammatory disease and nonspecific vaginosis; she received specific treatment but the IUD was not extracted (**Fig. 1,Fig. 2**).

At a second follow up visit – 12 weeks after the insertion of levonorgestrel-releasing intrauterine system no other patient was diagnosed with pelvic inflammatory disease.

## Discussion

 The results of our study are similar to those of Milton et al., Zorlu et al. and Walsh et al. – antibiotic prophylaxis has no effect on the incidence of pelvic inflammatory disease [**3**,**13**,**14**]. 

 Multiple studies demonstrated that the risk of pelvic inflammatory disease seems to be the highest in the first 3 weeks, due to the contamination of the upper genital tract during the insertion of the IUD [**3**,**15**,**16**]. We incriminate the same mechanism due to the fact that only one patient from the 44 evaluated subjects was diagnosed with acute pelvic inflammatory disease, after only 14 days past the insertion of levonorgestrel-releasing intrauterine system, a participant from group A, who received a prophylactic antibiotic. Also, at the follow up visit – 12 weeks after the insertion of IUD no other patient was diagnosed with pelvic inflammatory disease, suggesting the same, previously discussed mechanism of appearance of this redundant complication.

## Conclusion

Antibiotic prophylaxis is not mandatory, after the insertion of levonorgestrel-releasing intrauterine system in order to decrease the risk of pelvic inflammatory disease.

**Disclosure**: None of the authors have a conflict of interest.

**Acknowledgement**:This study was supported by the international project “Development of the translational research infrastructure in molecular and imagistic pathology - MOLIMAGEX”. Project manager: Prof. Catalin Cirstoiu, MD. Responsible for the Obstetrics-Gynecology Department: Assoc. Prof. Monica Cirstoiu, MD
